# Intensity of swimming exercise influences aortic reactivity in rats

**DOI:** 10.1590/1414-431X20154397

**Published:** 2015-09-18

**Authors:** A.F. Brito, A.S. Silva, I.L.L. Souza, J.C. Pereira, B.A. da Silva

**Affiliations:** 1 Programa de Pós-Graduação em Produtos Naturais e Sintéticos Bioativos, Centro de Ciências da Saúde, Universidade Federal da Paraíba, Paraíba, Brasil; 2 Laboratório de Farmacologia Funcional Professor George Thomas, Centro de Ciências da Saúde, Universidade Federal da Paraíba, Paraíba, Brasil; 3 Laboratório de Estudos do Treinamento Físico Aplicado ao Desempenho e Saúde, Departamento de Educação Física, Centro de Ciências da Saúde, Universidade Federal da Paraíba, Paraíba, Brasil

**Keywords:** Aerobic exercise, Anaerobic exercise, Lipid peroxidation, Smooth muscle, Swimming, Vessel

## Abstract

Exercise is known to cause a vasodilatory response; however, the correlation between the vasorelaxant response and different training intensities has not been investigated. Therefore, this study evaluated the vascular reactivity and lipid peroxidation after different intensities of swimming exercise in rats. Male Wistar rats (aged 8 weeks; 250-300 g) underwent forced swimming for 1 h whilst tied to loads of 3, 4, 5, 6, and 8% of their body weight, respectively (groups G3, G4, G5, G6 and G8, respectively; n=5 each). Immediately after the test, the aorta was removed and suspended in an organ bath. Cumulative relaxation in response to acetylcholine (10^−12^-10^−4^ M) and contraction in response to phenylephrine (10^−12^-10^−5^ M) were measured. Oxidative stress was estimated by determining malondialdehyde concentration. The percentages of aorta relaxation were significantly higher in G3 (7.9±0.20), G4 (7.8±0.29), and G5 (7.9±0.21), compared to the control group (7.2±0.04), while relaxation in the G6 (7.4±0.25) and G8 (7.0±0.06) groups was similar to the control group. In contrast, the percentage of contraction was significantly higher in G6 (8.8 ±0.1) and G8 (9.7±0.29) compared to the control (7.1±0.1), G3 (7.3±0.2), G4 (7.2±0.1) and G5 (7.2±0.2%) groups. Lipid peroxidation levels in the aorta were similar to control levels in G3, G4 and G5, but higher in G6 and G8, and significantly higher in G8 (one-way ANOVA). These results indicate a reduction in vasorelaxing activity and an increase in contractile activity in rat aortas after high-intensity exercise, followed by an increase in lipid peroxidation.

## Introduction

Improvements in vasomotor function are one of the many benefits associated with aerobic exercise. This involves an increase in endothelium-dependent vasodilation and an attenuation of the vasoconstrictor response, as observed in healthy animals (1-13), models of hypertension (4) and in humans (5,6).

Bechara et al. (7) showed that improvements in the vasodilator response occurred immediately after a single exercise session performed on a treadmill at 60% of maximal exercise capacity, directly related to an increase in the bioavailability of endothelial nitric oxide and attenuation of the maximal contractile response (8,9). This post-exercise response model is relevant to the development of post-exercise hypotension in humans, in which both hypertensive and normotensive subjects experience a reduction in blood pressure during the first minute after a single exercise session (10-12), which may continue for hours after the exercise session (13). This post-exercise hypotension may be partly explained by a transient increase in vasodilator activity (14).

Post-exercise hypotension is more evident following moderate-intensity exercise (60-70% of $\dot{\text{V}}$O_2_) (15), though even mild exercise has been reported to promote post-exercise hypotension (16,17). In contrast, high-intensity exercise has been reported to eliminate the hypotensive effect or even promote increased blood pressure (16,18). The mechanisms responsible for these changes in blood pressure in response to high-intensity exercise have been investigated and include roles for sympathetic activity (10), increased angiotensin-converting enzyme (19), and oxidative stress (20). However, no studies have yet determined if vascular reactivity is involved in the differential blood pressure response to exercise intensity.

We hypothesized that high-intensity exercise might result in a smaller relaxant response and greater contractile response in Wistar rats. Therefore, we evaluated the relaxation and contraction responses of the aorta in Wistar rats immediately after different intensities of swimming exercise and investigated the possible role of exercise-induced lipid peroxidation in these responses.

## Material and Methods

### Animals

Male Wistar rats (aged 8 weeks; 250-300 g) were obtained from the vivarium Prof. Thomas George (Centro de Biotecnologia, Universidade Federal da Paraíba). The animals were kept in ventilated cages with a balanced diet of feed pellets (Labina¯, Purina - Paulínia, Brasil) with access to water *ad libitum*, at constant temperature (21±1°C), and a daily 12-h light-dark cycle. The exercise tests were performed from 8-9 am and the cumulative concentration-response curves were obtained from 9 am - 10 pm. This study was performed in accordance with the Guide for the Care and Use of Laboratory Animals (1996). The study protocol was approved by the Ethics Committee for Animal Use of the Centro de Biotecnologia from the Universidade Federal da Paraíba (protocol #1101/11).

### Drugs and chemicals

Calcium chloride dihydrate (CaCl_2_.2H_2_O), magnesium chloride hexahydrate (MgCl_2_.6H_2_O), potassium chloride (KCl), and sodium bicarbonate (NaHCO_3_) were purchased from VETEC (Brazil). Monosodium phosphate 1-hydrate (NaH_2_PO_4_.H_2_O), glucose (C_6_H_12_O_6_), magnesium sulfate monohydrate (MgSO_4_.H_2_O), and hydrochloric acid (HCl PA) were purchased from Nuclear (Brazil). Sodium chloride (NaCl) was purchased from Dinâmica (Brazil). Carbamylcholine chloride, acetylcholine (ACh), and phenylephrine (Phe) were purchased from Sigma-Aldrich (USA). The carbogenic mixture (95% O_2_ and 5% CO_2_) was purchased from White Martins (Brazil).

### Equipment

Organ baths (5 mL) were heated to the appropriate temperature using thermostatic pumps (Fisatom 597; Fisatom, Brazil, and Polystat 12002; Cole-Parmer, USA), connected to a force transducer (TIM-50). These were connected to an amplifier (AECAD04F; both AVS Projetos, Brazil), which in turn was connected to a plate/D converter installed on a microcomputer with the AQCAD program version 8.0.5 (ANCAD version 5.33, Brazil).

### Exercise program

The animals were randomly divided into 6 groups of 5 rats each. Rats in the exercise groups were submitted to swimming sessions at five different exercise intensities (G3, G4, G5, G6 and G8) (see below) for 60 min, respectively. Rats in the control group (CG) were maintained under the same conditions as the exercised animals and were acclimatized on the experiment day, but did not perform the swimming tests.

According to Gobatto et al. (21), control rats were maintained in shallow clean water at 31±1°C throughout the entire experimental period and were euthanized at the end of the experiment.

Behaviors, including the tendency to adopt passive strategies (‘immobilization time') represented by sitting at the bottom of the tank, standing immobile on the bottom of the tank, floating, or carrying out small movements to keep their head above water, were observed by a trained researcher. Animals underwent two adaptation sessions 1 week before the experimental protocol, with an interval of 48 h between them, to minimize stress. The rats were put into the swimming tank for 30 min, with no additional load (22). Their behavior was analyzed during the first 15 min, which was the duration recorded in the forced swimming tests (23). Animals that failed to behave within 15 min were excluded, while animals that performed behaviors within this period were randomized among the exercise groups.

The swimming protocol was adapted from Chies et al. (1). We used a rectangular polyethylene tank (120-cm long × 50-cm deep x 43-cm wide) with water at 29±1°C. The rats were submitted to swimming exercise for a period of 60 min, with a metal ring attached to their chest by an elastic ribbon 1-cm wide. The ribbon was adjusted to fit the animal to prevent discomfort or stress and to avoid limiting the animal's movement during exercise. If the ribbon became displaced, the researcher paused the experiment, adjusted the ribbon, and then resumed the exercise test. The metal rings in groups G3, G4, G5, G6 and G8 represented 3, 4, 5, 6, and 8% of the animal's body weight, respectively, corresponding to a range of exercise intensities. The increase from 6% to 8% was based on the premise proposed by Gobatto et al. (21), who showed that, in rats submitted to different intensities of swimming exercise, 6% load corresponded to the maximal lactate steady state, while 8% represented higher exercise intensity. This study therefore compared intensities below the anaerobic threshold (3% and 4%), around the anaerobic threshold (5% and 6%), and above the threshold (8%).

### Assessment of exercise-induced lactate

Immediately at the end of the exercise sessions, 25 µL of arterial blood were withdrawn from the tail vein into calibrated heparinized capillaries. The samples were then placed in Eppendorf tubes containing 400 µL 4% trichloroacetic acid and refrigerated until analysis. Serum lactate levels were analyzed according to the protocol proposed by Engel and Jones (24).

### Organ preparation and aorta responsiveness

Five minutes after the end of exercise, rats were euthanized by cervical dislocation. Thoracic aortic rings 3-5 mm long were obtained free of connective tissue. The isometric response was determined by suspending individual rings by a stainless steel strap in organ baths (5 mL) containing Krebs solution (118.0 mM NaCl, 4.6 mM KCl, 5.7 mM MgSO_4_, 1.1 mM KH_2_PO_4_, 2.5 mM CaCl_2_, 11.0 mM glucose; 25.0 mM NaHCO_3_), adjusted to pH 7.4 and maintained at 37°C. The preparations were stabilized for 1 h under a resting tension of 1 g, and aerated with a mixture of 95% O_2_ and 5% CO_2_.

After the stabilization period, isometric contraction was induced by Phe 3×10^−7^ M. During the tonic phase of the contraction, Ach 10^−6^ M was added to verify the integrity of the endothelium (25). The vascular endothelium was considered complete when the aortic rings showed relaxation ≥50% (26), and non-functional if relaxation was ≤10%. After washing, the tonic component of a second reaction was induced by Phe 3×10^−7^ M for 30 min, followed by the cumulative addition of ACh to the organ baths (10^−12^-10^−4^ M) to induce relaxation.

For pharmacological evaluation of contractile vascular reactivity, the aorta was stabilized for 60 min and checked for the presence or absence of functional endothelium, as described above. A cumulative curve was then obtained by adding increasing concentrations of Phe (10^−12^-10^−5^M) to the organ bath. Aorta responsiveness was evaluated by comparing pD_2_ (negative logarithm of molar concentration of an agonist that produces 50% of its maximal effect) and E_max_ (maximum effect) values for the control and trained groups.

### Exercise-induced oxidative stress in the aorta and heart

The rates of lipid peroxidation in the aorta and heart were estimated by determining malondialdehyde (MDA), using the thiobarbituric acid test. Tissues were washed with cold saline to minimize interference of hemoglobin with free radicals and to remove adhered blood. They were then weighed and homogenized with KCl 10%, 250 µL was removed and warmed in a water bath at 37°C for 1 h, and 400 µL of perchloric acid 35% was added and centrifuged at 0.02 *g* for 20 min at 4°C. The supernatant was removed and placed in contact with 400 µL of thiobarbituric acid 0.6% and the mixture was incubated at 95-100°C for 1 h. After cooling, the absorbance of the supernatant was read at 532 nm. A standard curve was generated using 1,1,3,3-tetrametoxipropane. The results are reported as nmol MDA/mg protein. Protein concentration was measured using the Bradford method (27). The MDA concentration in each tissue sample was replaced by the absorbance values of the MDA standard curve obtained from different concentrations of a standard solution. The data were normalized by the dry weight in a given volume of the sample, where the absorbance values were divided by the weight in grams of the tissue.

### Statistical analysis

Data are reported as means±SE and were tested for normality and homogeneity using the Shapiro-Wilk and Levine tests, respectively. Comparisons between groups were made using one-way ANOVA, with *post hoc*Bonferroni's test. Values of P<0.05 were considered to be significant. The values of pD_2_ and E_max_ were calculated by non-linear regression. All results were analyzed using GraphPad Prism version 5.01 (GraphPad Software Inc., USA).

## Results

### Assessment of exercise-induced lactate

**Figure 1:**
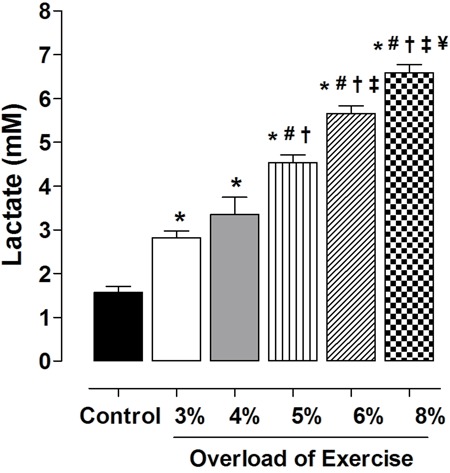
Lactate production as a function of exercise intensity. Data are reported as means±SE (n=5 per group). Exercise intensity was based on loading with 3, 4, 5, 6 and 8% of body weight, respectively, during swimming exercise. *P<0.05 *vs* control; ^#^P<0.05 *vs* 3%; ^+^P<0.05 *vs* 4%; ^‡^P<0.05 *vs* 5%; ^¥^P<0.05 *vs* 6% (one-way ANOVA).

Lactate content increased in proportion to the exercise intensity. There was no significant difference in lactate production between G3 (2.8±0.35 mM) and G4 (3.35±0.80 mM), but lactate production increased significantly with subsequent increases in exercise intensity: G5 > G4 (4.54±0.39 *vs* 3.35±0.80 mM, P<0.05); G6 > G5 (5.66±0.39 *vs* 4.54±0.39 mM, P<0.05); and G8 > G6 (6.59±0.42 *vs* 5.66±0.39 mM, P<0.05) (Figure 1).

### Pharmacological evaluation of rat aorta responsiveness

**Figure 2:**
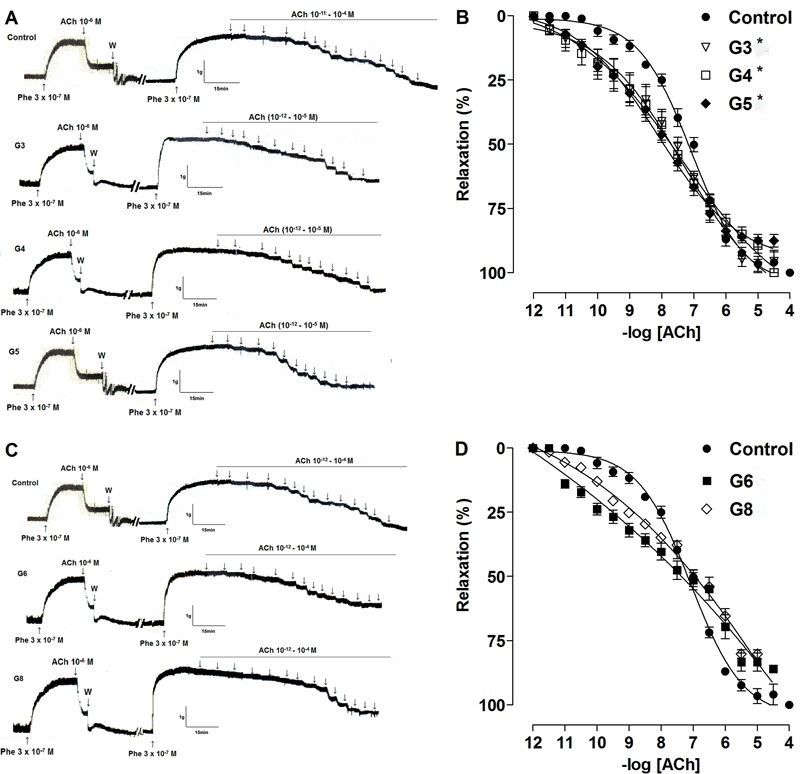
Representative traces and relaxant effect on rat aorta induced by acetylcholine in control, G3 G4, and G5 (*A* and *B*, lower intensities), and G6 and G8 (*C* and *D*, higher intensities) groups. Data are reported as means±SE (n=5 per group). G3, G4, G5, G6 and G8: exercise intensity based on loading with 3, 4, 5, 6 and 8% of body weight, respectively, during swimming exercise. *P<0.05 *vs* control (one-way ANOVA). Phe: phenylephrine; W: wash.

Aorta relaxation was significantly greater in G3, G4 and G5 compared to the CG (Figure 2A and B), as demonstrated by the values of pD_2_ (G3=7.9±0.20, G4=7.8±0.29, G5=7.9±0.21, CG=7.2±0.04, P<0.05). Conversely, aorta relaxation was similar to the control level in G6 and G8 (G6=7.4±0.25, G8=7.0±0.06) (Figure 2C and D). G8 showed reduced responsiveness compared to G3, G4 and G5.

E_max_(%) was significantly reduced in G6 and G8 compared to CG, G3, G4 and G5: CG=99±0.5; G3=98±3; G4=99±0.2; G5=97±0.84 *vs* G6=83±4 and G8=80±2 (P<0.05).

**Figure 3:**
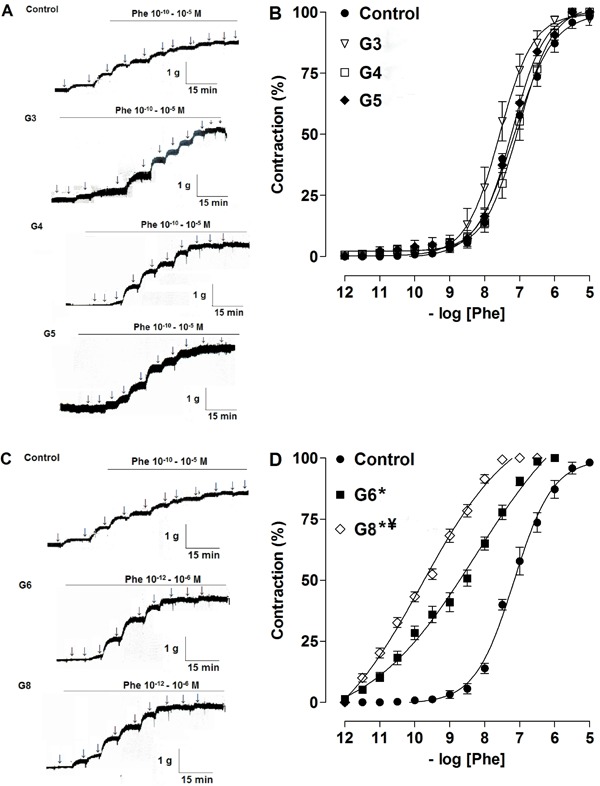
Representative traces and contractile response of rat aorta induced by phenylephrine in control, G3, G4, G5 (*A* and *B*, lower intensities), and G6 and G8 (*C* and *D*, higher intensities) groups. Data are reported as means±SE (n=5 per group). G3, G4, G5, G6 and G8: exercise intensity based on loading with 3, 4, 5, 6 and 8% of body weight, respectively, during swimming exercise. *P<0.05 *vs* control; ^¥^P<0.05 *vs* G6 (one-way ANOVA). Phe: phenylephrine.

The percentage of contraction of the aorta in response to exercise of different intensities varied. The contractile response to Phe was similar in G3, G4, G5 and CG (Figure 3A and B), but contraction at lower concentrations of Phe was significantly increased in G6 and G8 compared to CG, G3, G4, and G5 as demonstrated by pD_2_: CG=7.1±0.1; G3=7.3±0.2; G4=7.2±0.1; G5=7.2±0.2 *vs*G6=8.8±0.1; G8=9.7±0.29 (P<0.05). Furthermore, the aorta contractile response was significantly higher in G8 rats compared to G6 rats (9.7±0.29 *vs* 8.8±0.1, P<0.05) (Figure 3C and D).

### Exercise-induced oxidative stress in aorta and heart

**Figure 4:**
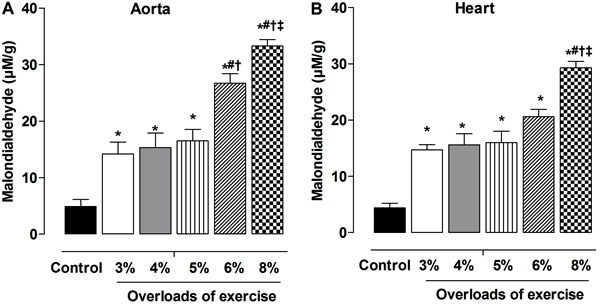
Levels of lipid peroxidation in aorta (*A*) and heart (*B*) in control group and groups with exercise intensity based on loading with 3, 4, 5, 6 and 8% of body weight, during swimming exercise. Data are reported as means±SE (n=5 per group). *P<0.05 *vs* control; ^#^P<0.05 *vs* 4%; ^+^P<0.05 *vs* 5%; ^‡^P<0.05 *vs* 6% (one-way ANOVA).

All the exercise protocols increased lipid peroxidation compared to controls, irrespective of the exercise intensity. Exercise intensity promoted lipid peroxidation (Figure 4). Lipid peroxidation in the aorta was similar in G3, G4 and G5, but increased in G6, and further increased in G8. Peroxidation in the heart was similar in G3, G4, G5 and G6, but significantly higher in G8.

## Discussion

The present study demonstrated that relatively low intensity swimming exercise (with a load of 3%, 4% or 5% of body weight) promoted acute vasorelaxant activity in the aorta without affecting its pattern of contraction. However, increased exercise intensity (load of 6% or 8% of body weight) reduced the relaxant effect of the exercise and increased the contractile response to Phe. The vascular response to loads of 6% or 8% was accompanied by a significant increase in lipid peroxidation.

We identified the metabolic demands of the exercise protocols by measuring lactate production, and noted that even the lowest intensity exercise increased lactacidemia. Exercise in the G3, G4 and G5 groups demonstrated aerobic characteristics, as proposed by Gobatto et al. (21) who identified lactate production of up to 5.5 mM as indicating aerobic predominance. However, our data differed slightly from those of Gobatto et al. (21), who reported that it was possible to exercise for about 30 min at the boundary between aerobic and anaerobic demands (equivalent to 5.5 mM in rats). In our study, rats that swam for 60 min had 6.59 mM lactate, which was incompatible with anaerobic activity. It is possible that our protocol merely reflected variations already reported in the literature. Carr et al. (28) reported an anaerobic threshold of 7 mM in human athletes, whereas the general consensus is that the anaerobic threshold occurs at 4 mM. We, therefore, interpreted our results in terms of increasing exercise intensity.

Most studies investigating the influence of exercise on vascular reactivity in animal models have assessed responses to chronic training. However, recent studies have also examined the vasorelaxant response to acute exercise. These studies demonstrated that acute exercise improved the vasorelaxant response by increasing the influx of calcium and the release of nitric oxide (29,30), and may also be mediated by insulin-like growth factors and by reduced superoxide production (31).

Vascular reactivity has been shown to differ between normotensive and hypertensive animals (4). Healthy animals in the exercise groups had pD_2_ values of about 7.60±0.11, compared to 6.96±12.19 in the control group. In hypertensive animals, ACh-induced relaxation produced pD_2_ values around 9.8±0.006 in the exercise group, compared to 8.7±0.1 in the control group.

Our results showed that the reduced relaxation associated with higher-intensity exercise (load of 6% or 8%) was simultaneously accompanied by a significant increase in the contractile response. This increased vasoconstrictor response may be mediated by sympathetic stimulation. Jendzjowsky and Delorey (32) reported that high-intensity treadmill exercise significantly increased sympathetic stimulation in the rat femoral artery, resulting in a significant increase in the magnitude of vasoconstriction compared to moderate-intensity exercise. The greater the stimulation frequency, the greater increase in sympathetic activity.

Studies in humans demonstrated that this increase in sympathetic activity was responsible for changes in blood pressure during high-intensity exercise (33). However, it is difficult to investigate the mechanisms in detail in humans, and the current study provides valuable information to help explain the increase in blood pressure during high-intensity exercise through an increase in the vasoconstrictor response.

In addition to increased blood pressure caused by vasoconstriction, our hypothesis suggests that increased oxidative stress during high-intensity exercise (20) may also help to minimize blood pressure reduction after exercise. This was supported by the fact that vasoconstriction associated with high-intensity exercise was accompanied by increased MDA production. Increased vasoconstrictor activity is activated by a reduction in nitric oxide bioavailability as a result of increased ROS production through NADPH oxidase activity. This in turn is associated with local release of angiotensin II and vasoconstrictor prostanoids, which contribute to the increase in peripheral resistance, vascular damage caused by endothelial oxidative stress, and increased blood pressure (20).

Interestingly, previous studies have used progressive exercise protocols in which animals performed increasing intensities of exercise until they reached fatigue (0.25-0.65 m/s). Although these protocols have been shown to be effective for observing differences in vascular reactivity in intense situations, they are less relevant to the situation in humans, where long-term, constant-intensity exercise protocols are adopted to prevent and treat cardiometabolic diseases. We, therefore, used an equivalent protocol in our current study. To the best of our knowledge, this study represents the first assessment of acute vascular reactivity responses in an animal model following a training protocol equivalent to that performed by humans.

In terms of exercise, moderate exercise is generally believed to be the most effective means of lowering blood pressure in humans, via reduced sympathetic activity, blood volume, and peripheral vascular resistance, and increased production of nitric oxide and peripheral blood flow. The results of the current study confirmed that vessels were more reactive to relaxing agents and less reactive to contractile agents in response to moderate-intensity exercise, affirming the role of vascular reactivity in post-exercise hypotension. The results, therefore, confirmed that moderate exercise is the most suitable intensity for promoting vasodilation as a direct determinant of post-exercise hypotension. Previous data demonstrated the involvement of sympathetic nerve activity in the pressure response after exercise, while the results of the present study suggest that lipid peroxidation may also help to regulate the pressure response after exercise. In conclusion, this study demonstrated that vascular reactivity participated in the differential blood pressure response in relation to exercise intensity.
